# Cardioprotective Effects of n-3 Polyunsaturated Fatty Acids: Orchestration of mRNA Expression, Protein Phosphorylation, and Lipid Metabolism in Pressure Overload Hearts

**DOI:** 10.3389/fcvm.2021.788270

**Published:** 2022-01-03

**Authors:** Xiang Li, Weijiang Tan, Shuang Zheng, Junjie Zhang, Caiyi Zhu, Chun Cai, Honghua Chen, Chenqi Yang, Le Kang, Zhanhong Pan, W. Glen Pyle, Peter H. Backx, Yunzeng Zou, Feng Hua Yang

**Affiliations:** ^1^Guangdong Laboratory Animals Monitoring Institute, Guangzhou, China; ^2^College of Veterinary Medicine, South China Agricultural University, Guangzhou, China; ^3^School of Pharmacy, Guangdong Medical University, Dongguan, China; ^4^Faculty of Arts and Sciences, University of Toronto, Toronto, ON, Canada; ^5^Shanghai Institute of Cardiovascular Diseases, Zhongshan Hospital and Institutes of Biomedical Sciences, Fudan University, Shanghai, China; ^6^Department of Biomedical Sciences, University of Guelph, Guelph, ON, Canada; ^7^Department of Physiology, University of Toronto, Toronto, ON, Canada; ^8^Department of Biology, York University, Toronto, ON, Canada

**Keywords:** n-3 PUFA, pressure overload, dilated cardiomyopathy cardioprotection, mitochondrial function, multi-omics analysis

## Abstract

**Background:** Pressure overload can result in dilated cardiomyopathy. The beneficial effects of n-3 polyunsaturated fatty acids (n-3 PUFAs) on heart disorders have been widely recognized. However, the molecular mechanisms underlying their protective effects against cardiomyopathy remain unclear.

**Methods:** Pressure overload in mice induced by 8 weeks of transverse aortic constriction was used to induce dilated cardiomyopathy. A transgenic fat-1 mouse model carrying the n-3 fatty acid desaturase gene fat-1 gene from *Caenorhabditis elegans* was used to evaluate the mechanism of n-3 PUFAs in this disease. Echocardiography, transmission electron microscopy, and histopathological analyses were used to evaluate the structural integrity and function in pressure overloaded fat-1 hearts. mRNA sequencing, label-free phosphoprotein quantification, lipidomics, Western blotting, RT-qPCR, and ATP detection were performed to examine the effects of n-3 PUFAs in the heart.

**Results:** Compared with wild-type hearts, left ventricular ejection fraction was significantly improved (C57BL/6J [32%] vs. fat-1 [53%]), while the internal diameters of the left ventricle at systole and diastole were reduced in the fat-1 pressure overload hearts. mRNA expression, protein phosphorylation and lipid metabolism were remodeled by pressure overload in wild-type and fat-1 hearts. Specifically, elevation of endogenous n-3 PUFAs maintained the phosphorylation states of proteins in the subcellular compartments of sarcomeres, cytoplasm, membranes, sarcoplasmic reticulum, and mitochondria. Moreover, transcriptomic analysis predicted that endogenous n-3 PUFAs restored mitochondrial respiratory chain function that was lost in the dilated hearts, and this was supported by reductions in detrimental oxylipins and protection of mitochondrial structure, oxidative phosphorylation, and ATP production.

**Conclusions:** Endogenous n-3 PUFAs prevents dilated cardiomyopathy via orchestrating gene expression, protein phosphorylation, and lipid metabolism. This is the first study provides mechanistic insights into the cardioprotective effects of n-3 PUFAs in dilated cardiomyopathy through integrated multi-omics data analysis.

## Introduction

Pressure overload can be induced by hypertension or aortic valve stenosis. It has been estimated that 31.1% of adults have hypertension globally (1), whereas the prevalence of severe aortic stenosis in those aged 75 years and older is 3.4% in European countries and North America (2). Long-standing pressure overload leads to hypertrophic remodeling of the heart and can progress to dilated cardiomyopathy (DCM). This advanced stage of heart disease is characterized by dilation of the atria and ventricles as well as reduced systolic and diastolic function (3). Mutations in genes encoding myofilaments [e.g., titin (4), myosin heavy chain (5), and myosin binding protein C (6)], as well as mitochondrial proteins (7) have been frequently reported in studies of inherent DCM. Systematic changes, including activation of the renin angiotensin-aldosterone system (8), metabolic disorder (9), microvascular injury (10), fibrosis and inflammation (11, 12), and activation of intracellular signaling (13, 14) were detected in pressure overload-induced dilated myocardia. Our pervious study demonstrated that myofilament phosphorylation is a crucial regulatory event in failing hearts (15, 16). Treatment strategies involving angiotensin-converting enzyme inhibitors, device therapy, and etiology-based therapies are used to control blood pressure, restore normal heart rhythm, lower inflammation, and prevent blood clot formation in hypertension-associated cardiomyopathy (3). However, effective treatments for cardiomyopathy involving dilated chambers are lacking.

N-3 polyunsaturated fatty acids (n-3 PUFAs) are important components of cellular membranes. The beneficial effects of n-3 PUFAs on cardiovascular disease have been demonstrated in epidemiological and clinical studies (17, 18). However, the human body cannot synthesize n-3 PUFAs with odd numbers of carbon atoms. Supplementation with n-3 PUFAs including eicosatetraenoic acid (EPA) and docosahexaenoic acid (DHA) has long been performed as a simple alternative approach for blood pressure management. N-3 PUFAs can suppress aldosterone secretion, lower systemic vascular resistance, and reduce blood pressure (19). Metabolism of EPA and DHA share the same pathways with arachidonic acid (AA) including cyclooxygenase (COX), 5-lipoxygenase (LOX), and cytochrome P450 monooxygenase (CYP) enzymes (20). Endogenous n-3 PUFA-derived oxygenated metabolites of EPA and DHA exert beneficial roles. Moreover, n-3 PUFAs show potential for improving cardiac function attributed to pressure overload; however, as the treatment of patients with hypertension is often combined with other medications or device intervention, the molecular mechanisms associated with the beneficial effects of these unsaturated fatty acids in cardiomyopathy remain unclear. Here, we investigated molecular signatures including mRNA expression, protein phosphorylation, and lipid metabolism in a murine pressure overload model to aid to our understanding of the effects elicited by n-3 PUFAs against dilated cardiomyopathy. We further explored whether n-3 PUFAs regulate mitochondrial function that were potentially involved in cardioprotection.

## Materials and Methods

### Animals and Experimental Design

Fat-1 mice were developed by the Kang Laboratory (21). These mice expressed the n-3 fatty acid desaturase gene *fat-1* from *Caenorhabditis elegans*, which converts n-6 PUFAs into n-3 PUFAs, resulting in high levels of endogenous n-3 PUFAs in the heart. Wild-type (WT) and transgenic fat-1 littermates (fat-1) aged 3 months were subjected to a transverse aortic constriction (TAC; the surgical procedure is described below) to develop pressure overload. The four groups of animals were named as WT-Sham, Fat1-Sham, WT-TAC, and Fat1-TAC. After the experiments, the cardiac function of the animals was evaluated using echocardiography. The heart tissues were collected for histological examination, transmission electron microscopy (TEM), mRNA sequencing, label-free protein quantification, lipidomics, and western blotting were conducted to explore effects and underlying mechanisms of the elevation of endogenous n-3 PUFAs in dilated cardiomyopathy.

All animal experiments were conducted in accordance with the guidelines of the Institutional Animal Care and Use Committee of the Guangdong Laboratory Animals Monitoring Institute (No. IACUC2017015). The mice were housed in a specific pathogen-free mouse facility. The ambient temperature and humidity of the facility were 24 ± 2°C and 40–60%, respectively, with a 12-h light-dark cycle. The animals were given *ad libitum* access to water and a regular rodent diet (Keao Xieli Feed Co., Beijing, China). The animal facility was accredited by the Association for Assessment and Accreditation of Laboratory Animal Care.

### Transverse Aortic Constriction

To introduce pressure overload stress to the heart, TAC was performed in WT and fat-1 mice. After anesthesia, the mice were intubated and connected to a ventilator. Isoflurane (1–2%) was administered to maintain anesthesia. A 1-cm incision was made on the chest midline around the second rib to open the thoracic cavity. After identifying the aortic thoracic segment, a 7-0 suture was installed under the aorta in the brachiocephalic trunk and the left common carotid artery, and a 26G blunt needle was inserted parallel to the aorta. The needle was removed after the surgical knot was tied. The sham groups of WT and fat-1 mice were subjected to the open chest procedure but not aortic constriction.

### Echocardiography

An ultrasound system equipped with a high-frequency probe MS-440 (Vevo 2100; VisualSonics, Toronto, ON, Canada) was used to evaluate the structure and function of WT and fat-1 mice with ventricular pressure overload. Horizontal parasternal short axis B-mode and M-mode images of the heart were recorded for each mouse group (WT-Sham, WT-TAC, Fat1-Sham, and Fat1-TAC). B-mode images of the parasternal short axis in the papillary muscle plane were obtained by placing the probe on the chest midline. M-mode images were acquired at the same position as those of the B-mode recording. The echocardiographic indices included interventricular septum thickness at end-systole and diastole (IVS;s and IVS;d), left ventricular posterior wall thickness at end-systole and diastole (LVPW;s and LVPW;d), left ventricular internal dimension at end-systole and diastole (LVID;s and LVID;d), left ventricular ejection fraction (EF), and short axis fractional shortening (FS).

### Histological Examination

Pathological examinations were conducted to determine the cardiac structure, fibrosis, vessel morphology, and cardiomyocyte size as previously described (15, 22). Masson's trichrome staining was performed to identify collagen fibers and muscle tissue, whereas wheat germ agglutinin was used to visualize cardiomyocyte plasma membranes and calculate the cardiomyocyte size. Mice from all four groups (WT-Sham, WT-TAC, Fat1-Sham, and Fat1-TAC) were euthanized, after which their hearts were excised, fixed for 12–16 h in 4% (v/v) paraformaldehyde, embedded in paraffin, and sectioned into 3-μm-thick slices. The following chemicals were used for histological examination: 4',6-diamidino-2-phenylindole (No. P36962; Invitrogen, Carlsbad, CA, USA), Bouin's solution (No. HT10132; Millipore, Billerica, MA, USA) and Weigert's hematoxylin (No. HT10132; Millipore), Biebrich scarlet-acid fuchsin solution (No. HT151; Millipore), and Aniline blue (No. B8653; Millipore). Pathological changes in pressure overload hearts were identified microscopically (DM2500; Leica Microsystems, Wetzlar, Germany). Cardiomyocytes were analyzed using ImageJ 1.52a software (NIH, Bethesda, MD, USA).

### Transmission Electron Microscopy

TEM was conducted to examine subcellular changes associated with n-3 PUFA expression. Left ventricular tissues were cut into small blocks (~1 mm^3^), fixed with 2.5% glutaraldehyde and 1% OsO_4_, dehydrated in ethanol, and embedded in Araldite. The tissue blocks were cut into slices with a thickness of 60 nm using a Leica cryostat system (EM UC7/FC7) and collected on copper grids. The ultrathin sections were double-stained with 3% uranyl acetate and lead citrate. The subcellular structure was observed using a Tecnai G2 Spirit transmission electron microscope (FEI Company, Hillsboro, OR, USA).

### mRNA Sequencing

Total RNA was extracted from the whole hearts of the four groups (WT-Sham, WT-TAC, Fat1-Sham, Fat1-TAC) using TRIzol^®^Reagent (Thermo Fisher Scientific). The RNA concentration and its integrity was assessed using an RNA Nano 6000 Assay Kit (Agilent Technologies, Santa Clara, CA, USA). A NEBNext^®^Ultra™ RNA Library Prep Kit for Illumina^®^(New England Biolabs, Ipswich, MA, USA) was used to generate sequencing libraries. Index codes were added, and the samples were clustered with a cBot Cluster Generation System using a TruSeq PE Cluster Kit v3-cBot-HS (Illumina, San Diego, CA, USA). An Illumina HiSeq platform (Illumina) was used to sequence the libraries.

Raw data was preprocessed for quality control, after which the clean reads were mapped to the reference transcriptome. Next, the four groups of processed data were compared in pairs, and the fold-changes between comparisons were log2-transformed. An adjusted *P*-value <0.1 was set as the cutoff for identifying differentially expressed genes (DEGs). DEGs with fold-changes >1.5 were considered as upregulated, whereas those with values <1/1.5 were considered downregulated.

### Phosphoproteomic Analysis

#### Filter-Aid Sample Preparation

The levels of phosphoprotein in the animal hearts (WT-Sham, WT-TAC, Fat1-Sham, and Fat1-TAC) were quantified using label-free quantitative proteomics technology. A filter-aid sample preparation protocol from Mann Laboratory was used to digest proteins and elute the peptides (23, 24). Briefly, total protein was extracted from the heart tissues using lysis buffer containing 4% sodium dodecyl sulfate (SDS), 100 mM Tris-HCl (pH 7.6), and 1 mM dithiothreitol, and the protein concentration was determined using a Pierce BCA Protein assay kit (No. 23225, Thermo Fisher Scientific, Waltham, MA, USA). The samples were desalted using an Empore SPE Cartridge C18 column (No. 98-0604-0198-5, Sigma, St. Louis, MO, USA; bed internal diameter, 7 mm; volume, 3 mL). The proteins were then concentrated and reconstituted in 40 μL of 0.1% formic acid (No. F0507, Sigma). The protein concentration was measured with a UV light a Spectrometer at 280 nm. Sample quality was verified by performing 12.5% SDS-PAGE protein separation and Coomassie Blue staining.

#### Liquid Chromatography-Tandem Mass Spectrometry

Phosphopeptides were enriched using a High-SelectTM Fe-NTA Phosphopeptides Enrichment Kit according to the manufacturer's instructions (No. A32992, Thermo Fisher Scientific). After lyophilizatoin, the phosphopeptides peptides were resuspended in 20 μL loading buffer (0.1% formic acid). For liquid chromatography-tandem mass spectrometry (LC-MS/MS) analysis, phosphopeptide samples were injected into a C18-reversed phase analytical column (Thermo Fisher Scientific Easy-Spray Column, No. ES900) and separated with a linear gradient over 120 min at a flow rate of 300 nL/min controlled by IntelliFlow technology. Spectra were acquired on a Q-Exactive orbitrap mass spectrometer (Thermo Fisher Scientific) coupled to an Easy-nLC 1200 system (Thermo Fisher Scientific). MS data were acquired from a survey scan (300–1800 m/z) for higher-energy C-trap dissociation fragmentation. The parameters were: automatic gain control 3e6, maximum inject time of 10 ms, and dynamic exclusion duration 40.0 s. Survey scans were acquired at a resolution of 70,000 at m/z 200, and the resolution for higher-energy C-trap dissociation spectra was set to 17,500 at m/z 200 and isolation width was 2 m/z. The normalized collision energy was 30 eV and the underfill ratio, which specifies the minimum percentage of the target value likely to be reached at maximum fill time, was defined as 0.1%. MS/MS spectra were searched using the MaxQuant 1.5.3.17 software for identification and quantitation analysis.

The label-free quantitative data for phosphoproteins from the four groups (WT-Sham, WT-TAC, Fat1-Sham, Fat1-TAC) were compared in pairs, and the fold-changes between comparisons were log2-transformed. A 0.05 cutoff was set for the *P*-value to identify differentially expressed phosphoproteins (DEPs). DEPs with fold-change values >1.5 and <1/1.5 were considered as upregulated and downregulated, respectively.

### Bioinformatic Analysis for mRNA and Phosphoprotein Expression

#### Biological Process Enrichment Analysis and Protein-Protein Interaction Network Construction

The web-accessible tool Metascape (https://metascape.org) was used to enrich biological processes for the datasets of DEGs and DEPs. Significant enrichment was considered as a *P*-value cutoff of 0.01, minimum overlap of 3, minimum enrichment of 1.5, and limiting-network interactome consisting of 3–500 candidate proteins. A high *P*-value (–log10) of the term in a biological process reflected its relatively higher degree of enrichment. Each DEG or DEP in the interaction network was displayed in the modules according to its biological processes.

Metascape was used to construct protein-protein interaction networks for the enrichment biological processes (25). Biological processes with similar functions were clustered, and then these enrichment clusters were connected according to the algorithm MCODE. Diagrams of the protein interaction network were visualized in Cytoscape v. 3.8.1 (http://www.cytoscape.org).

#### Venn Diagram Analysis for Differentially Expressed Phosphoproteins

To determine the roles of endogenous n-3 PUFAs in pressure overload hearts, each comparison pair was input into the TBtools program (https://omictools.com/tbtools-tool) to generate Venn diagrams (http://jvenn.toulouse.inra.fr). Here, two intersections were obtained: (1) the intersection of upregulated DEPs in the WT-TAC vs. WT-Sham comparison group and downregulated DEPs in the Fat1-TAC vs. WT-TAC comparison group, and (2) the intersection of downregulated DEPs in the WT-TAC vs. WT-Sham comparison group and upregulated DEPs in the Fat1-TAC vs. WT-Sham comparison group. Next, these two intersections further excluded the up-/downregulated DEPs from the Fat1-Sham vs. WT-Sham comparison. Finally, two new sets of DEPs were obtained: (1) upregulated by pressure overload in the WT hearts but downregulated by fat-1 transgene in the pressure overload condition, and (2) downregulated by pressure overload in the WT hearts but upregulated by fat-1 transgene under pressure overload.

#### Mitochondrial Function Prediction

Venn diagram analysis was also used to identify genes associated with the mitochondrial respiratory chain complexes that were upregulated by pressure overload in WT hearts, but downregulated by fat-1 transgene in the pressure overload condition. Here, a *P*-value <0.05 was set as the cutoff for screening DEGs associated with the mitochondrial respiratory chain function.

### Lipidomic Analysis of n-3 and n-6 PUFAs

#### Reagents

Chemical used in this study included methanol (No.106009; Merck, Darmstadt, Germany), formic acid (No. 5438040; Merck), ethanoic acid (No. US-FLSA-001; Thermo Fisher Scientific), and ethyl acetate (No. E196SK-4, Thermo Fisher Scientific). The isotope internal standards (IS) for quantification of lipid oxylipins were obtained from Cayman Chemical (Ann Arbor, MI, USA), which are listed as follows: prostaglandin (PG) E2-d4 (No. 314010), PGD2-d4 (No. 312010), PGF2α-d4 (No. 10007275), 6-keto-PGF1α-d4 (No. 315210), leukotriene (LT) C4-d5 (No. 10006198), tetranor-PGEM-d6 (No. 314840), LTB4-d4 (No. 320110), 15-hydroxy-eicosatetraenoic acid (HETE)-d8 (No. 31746), 5-HETE-d8 (No. 334230), thromboxane (TX) B2-d4 (No. 319030),12-HETE-d8 (No. 31745), platelet-activating factor (PAF)-d4 (No. 360900), oleoylethanolamide (OEA)-d4 (No. 9000552), eicosapentaenoic acid (EPA)-d5 (No. 10005056), docosahexaenoic acid (DHA)-d5 (No. 10005057), and arachidonic acid (AA)-d8 (No. 390010). These standards were mixed with methanol to obtain a final concentration of 100 ng/mL.

#### Extraction of n-3 and n-6 Polyunsaturated Fatty Acids and Oxylipins

The hearts from each group of mice (WT-Sham, WT-TAC, Fat1-Sham, and Fat1-TAC, *N* = 4) were collected and snap-frozen in liquid nitrogen. Total lipids were extracted from the heart tissues using a liquid-liquid extraction protocol as described previously with modifications (26), and 100 mg of heart tissue was homogenized by adding 1 L of methanol (2% formic acid and 0.01 mol/L BHT) and IS mixture (10 μL) and processing in an Omni tissue homogenizer (TH02, Kennesaw, GA, USA). The mixtures were centrifuged at 12,000 × *g* at 4°C for 10 min to collect the supernatants, which were mixed with 700 mL of ultra-pure water and 1 mL of ethyl acetate. The mixtures were centrifuged again at 4000 × *g* at 4°C for 10 min, and the upper organic phase was collected. The organic phase was dried using a nitrogen blower.

#### Ultra-Performance Liquid Chromatography-Tandem Mass Spectrometry

The dried extracts were dissolved in 10% acetonitrile (No. 900686, Sigma), filtered through a 0.45-μm membrane filter (No. HAWP04700; Millipore), and subjected to ultra-performance liquid chromatography-MS/MS analysis. A Shimadzu LCMS-8050 (Kyoto, Japan) equipped with a Phenomenex C18 column (2.1 mm × 150 mm × 2.6 μm; Torrance, CA, USA) was used to determine the n-3 and n-6 PUFAs and their oxylipins in the heart tissue extracts. The column temperature was set at 40°C, and the injection volume was 5 μL. The mobile phase flow rate was 0.6 mL/min. Gradient elution was performed with 0.1% (v/v) formic acid (solvent A) and acetonitrile (solvent B). The gradient of mobile phase B was set as follows: 10% (0 min), 25% (5 min), 35% (10 min), 75% (20 min), 95% (20.1 min), 95% (25 min), 10% (25.1 min), and 10% (30 min).

#### Quantitative Analysis of Oxylipins

MS/MS analyses were conducted in both positive and negative ion modes using nitrogen as the nebulizer gas, and PUFAs and oxylipins were identified according to multiple reaction monitoring and relative retention time of the species in the same class. The mass spectrometer was operated with MS operating software (lipid mediator version 2 software package, Shimadzu).

The identified lipid oxylipins were quantified by measuring the peak areas of the detected species as previously described (26). Briefly, the peak areas of each individual lipid oxylipin were corrected by those corresponding to the ISs of the same lipid metabolite class, which compensated for the fluctuations in MS intensities observed during different runs. The corrected peak areas of all identified lipid oxylipins were used for in-depth analysis.

Lipidomic analysis was performed as described previously (16). To process the raw data, MetaboAnalyst V5.0 (Montreal, Canada) was used to generate the clustering diagram and SIMCA 14.1 (Umetrics, Umeå, Sweden) was used for principal component analysis, providing an overview of the dataset structure and relationships. A *P*-value <0.05 was set as the cutoff for identification of the significant changes of oxylipins between groups. Fold-change values >1.5 were considered as upregulated, whereas values <1/1.5 were considered as downregulated.

### Multi-Omics Data Analysis

To integrate multi-omics data and understand the insights of n-3 PUFA regulation in fat1-TAC hearts, comparison datasets (Fat1-TAC vs. WT-TAC) from RNA-sequencing, phosphoproteomics, or lipidomics were uploaded to MetaboAnalyst V5.0. The top 10 most enriched pathways were selected for further analysis.

### Real Time Quantitative Polymerase Chain Reaction

RT-qPCR was used to verify the expression of genes identified by multi-omics data analysis. Briefly, after total RNA extraction, TB Green Premix Ex Taq II (No. RR820; Takara Bio Inc., Shiga, Japan) was used according to manufacturer's instruction. The reaction steps included 95°C for 30 s, 40 cycles of 95°C for 5 s, 60°C for 34 s, and a final extension at 72°C for 10 min. Transcript levels were normalized to the expression of β-actin. The primers in this experiment listed in Supplementary Table S1.

### Western Blotting

Expression of fission (dynamin-related protein 1, DRP1) and fusion (optic atrophy-1, OPA1) proteins and oxidative phosphorylation markers (electron transport chain complexes) of the mitochondria was determined to evaluate mitochondrial function (27). The hearts were homogenized in cell lysis buffer (#9803, Cell Signaling Technology, Danvers, MA, USA) containing protease inhibitors and 1% Triton X-100. The samples were centrifuged at 12000 × *g* at 4°C for 15 min, and the supernatants were retained for further use. Cytosolic (40 μg) proteins were separated using 10% SDS-PAGE and transferred to polyvinylidene fluoride blotting membranes (Millipore). After blocking with 5% skim milk, the membranes were incubated with primary antibodies (as listed above) overnight at 4°C before being incubated with species-appropriate secondary antibodies (Cell Signaling Technology). The bands were detected with Immobilon Western Chemiluminescent HRP Substrate (Millipore). Primary antibodies against DRP1 (No. 8570S, Cell Signaling Technology) and OPA1 (No. 67589S, Cell Signaling Technology) were used, along with a total oxidative phosphorylation rodent western blotting antibody cocktail (No. ab110413, Abcam, Cambridge, UK). Band density was analyzed using ImageJ software (NIH).

### Adenosine Triphosphate Assay

The whole heart was homogenized and adenosine triphosphate (ATP) was quantitatively determined using an ATP colorimetric assay kit (#A095-1-1, Nanjing Jiancheng Co., Nanjing, China) following the manufacturer's instructions. Briefly, the homogenates of heart tissues (200 mg in 1.8 mL of milli-Q H_2_O) were heated to 100°C for 10 min and centrifuged at 1150 × *g* for 10 min. The amount of phosphorylating glycerol generated from the supernatants was read at 630 nm using a spectrophotometer (Ultrospec 2100, Biochrom, Cambridge, UK).

### Statistical Analysis

For echocardiographic and pathological examinations, the data are presented as the mean ± SEM. Raw data for the WT-Sham, WT-TAC, Fat1-Sham, and Fat1-TAC groups were uploaded to GraphPad Prism 8.0 software (GraphPad, Inc., La Jolla, CA, USA). Significant differences among multiple data groups were analyzed by two-way analysis of variance followed by Tukey's multiple comparisons test (GraphPad Prism 8.0). Graphs showing individual data points were plotted. For mRNA sequencing, phosphoproteomics, and lipidomics analyses, *t*-tests were used to identify significant differences between pairs of means. Statistical significance was set at *P* < 0.05.

## Results

### Elevation of Endogenous n-3 PUFAs Reduces Cardiac Remodeling and Improves Cardiac Function

**Figure 1:**
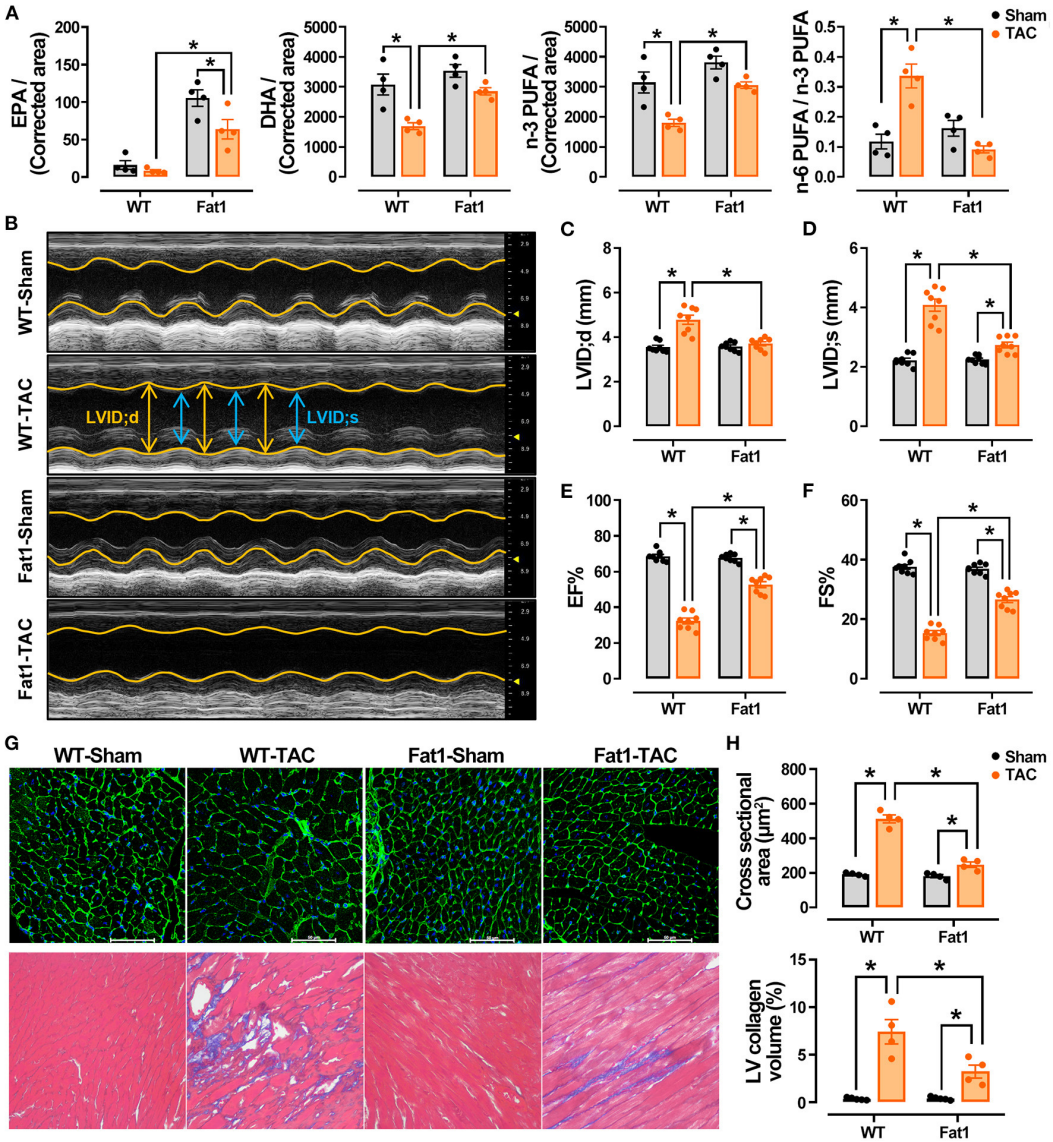
Endogenous n-3 PUFAs and echocardiographic measurements in WT and fat-1 mice with or without pressure overload. Fat-1 transgene increased endogenous n-3 PUFAs in normal and pressure overload hearts and lowered the ratio of n-6 to n-3 PUFAs in pressure overload hearts **(A)**. *N* = 4 for each group. EPA, eicosapentaenoic acid; DHA, docosahexaenoic acid; PUFA, poly unsaturated fatty acid. The M-mode recordings of the four groups are shown in **(B)**. Transverse aortic constriction (TAC)-induced pressure overload significantly increased the internal diameters at diastole **(C)** and systole **(D)** in WT mice, whereas these two indices were lower in fat-1 mice than in WT mice. Cardiac contractile function indices, including EF **(E)** and FS **(F)**, showed that the function was preserved in the fat-1 TAC group. Pressure overload significantly increased the size of cardiomyocytes and fibrosis, which was significantly reduced in the Fat1-TAC group **(G,H)**. *N* = 8 for each group, subjected to echocardiographic analysis, and *N* = 4 for each group subjected to histological observation. LV, left ventricle. All data are presented as the means ± SEM. ^*^P < 0.05 between groups.

We found that the levels of EPA were significantly increased in the hearts of fat-1 mice. After TAC, both the EPA and DHA levels were significantly higher in the myocardium (Figure 1A). The total levels of n-3 PUFAs were significantly higher in the fat-1 TAC hearts than in the WT-TAC hearts, whereas the levels of n-6 PUFAs and ratio of n-6/n-3 PUFA were significantly lower in the fat1-TAC hearts than in the WT-TAC hearts (Figure 1A). These measurements suggest that the fat-1 transgene increased endogenous n-3 PUFAs and in the normal and pressure overload hearts, as well as lowered the ratio of n-6 to n-3 in pressure overload hearts.

The heart rate did not differ between groups based on echocardiographic recording (Figure 1B; Table 1). TAC-induced pressure overload significantly increased LVID;s (*P* < 0.0001) and LVID;d (*P* < 0.0001) in WT mice, whereas these two indices were significantly lower in fat-1 mice than in WT mice (Figures 1B–D). Correspondingly, pressure overload significantly increased the LV volume at systole (*P* < 0.0001) and diastole (*P* < 0.0001) in WT mice. The corrected LV mass was significantly increased in WT mice but not in Fat-1 mice (Table 1). Cardiac contractile function indices, including EF and FS, showed that the function was preserved in the Fat-1 TAC group but not in the WT-TAC group (Figures 1B,E,F).

**Table 1:** Echocardiographic measurements.

**Parameters**	**WT-Sham**	**WT-TAC**	**Fat1-Sham**	**Fat1-TAC**
Heart rate (beats/min)	455 ± 6	440 ± 7	467 ± 10	450 ± 11
LV Mass (corrected), mg	102.43 ± 4.11	179.68 ± 16.43^*^	102.35 ± 4.44^#^	118.71 ± 5.42^#^
Interventricular ventricular septum thickness, end-systolic, IVS;s (mm)	1.36 ± 0.01	1.23 ± 0.02	1.34 ± 0.03	1.24 ± 0.03
Interventricular ventricular septum thickness, end-diastolic, IVS;d (mm)	0.89 ± 0.01	0.87 ± 0.02	0.87 ± 0.03	0.89 ± 0.04
Left ventricular posterior wall thickness, end-systolic, LVPW;s (mm)	1.12 ± 0.03	1.05 ± 0.02	1.18 ± 0.02^#^	1.17 ± 0.02^&^
Left ventricular posterior wall thickness, end-diastolic, LVPW;d (mm)	0.77 ± 0.02	0.88 ± 0.04^*^	0.77 ± 0.01^#^	0.86 ± 0.02
Left ventricular internal dimension, end-systolic, LVID;s (mm)	2.22 ± 0.07	4.08 ± 0.21^*^	2.24 ± 0.05^#^	2.74 ± 0.10^#^
Left ventricular internal dimension, end-diastolic, LVID;d (mm)	3.55 ± 0.08	4.78 ± 0.20^*^	3.58 ± 0.07^#^	3.71 ± 0.09^#^
Ventricular volume at systole (μL)	16.85 ± 1.28	74.91 ± 0.48^*^	17.53 ± 0.94^#^	27.99 ± 2.37^#^
Ventricular volume at diastole (μL)	53.36 ± 3.19	109.26.23 ± 10.37^*^	54.26.31 ± 0.20^#^	58.74 ± 3.53^#^
Ejection fraction, EF (%)	68.58 ± 1.04	32.35 ± 1.66^*^	67.71 ± 0.76^#^	52.73 ± 1.67*^#&^
Short axis fractional shortening, FS (%)	37.57 ± 0.82	15.33 ± 0.83^*^	36.89 ± 0.59^#^	26.58 ± 1.01*^#&^

*All data are presented as the means ± SEM. N = 8*.

* *P < 0.05 vs. WT-Sham*,

# *P < 0.05 vs. WT-TAC, and*

& *P < 0.05 vs. Fat1-Sham (two-way ANOVA, Tukey's multiple comparisons test)*.

In addition, we found that pressure overload significantly increased the size of cardiomyocytes and fibrosis (Figures 1G,H). Fibrosis was significantly reduced in the myocardium, and cardiomyocyte hypertrophy was reduced in the Fat1-TAC group (Figures 1G,H).

### Endogenous PUFAs Target Phosphorylation Signaling and Restore Protein Phosphorylation States Under Pressure Overload Conditions

**Figure 2:**
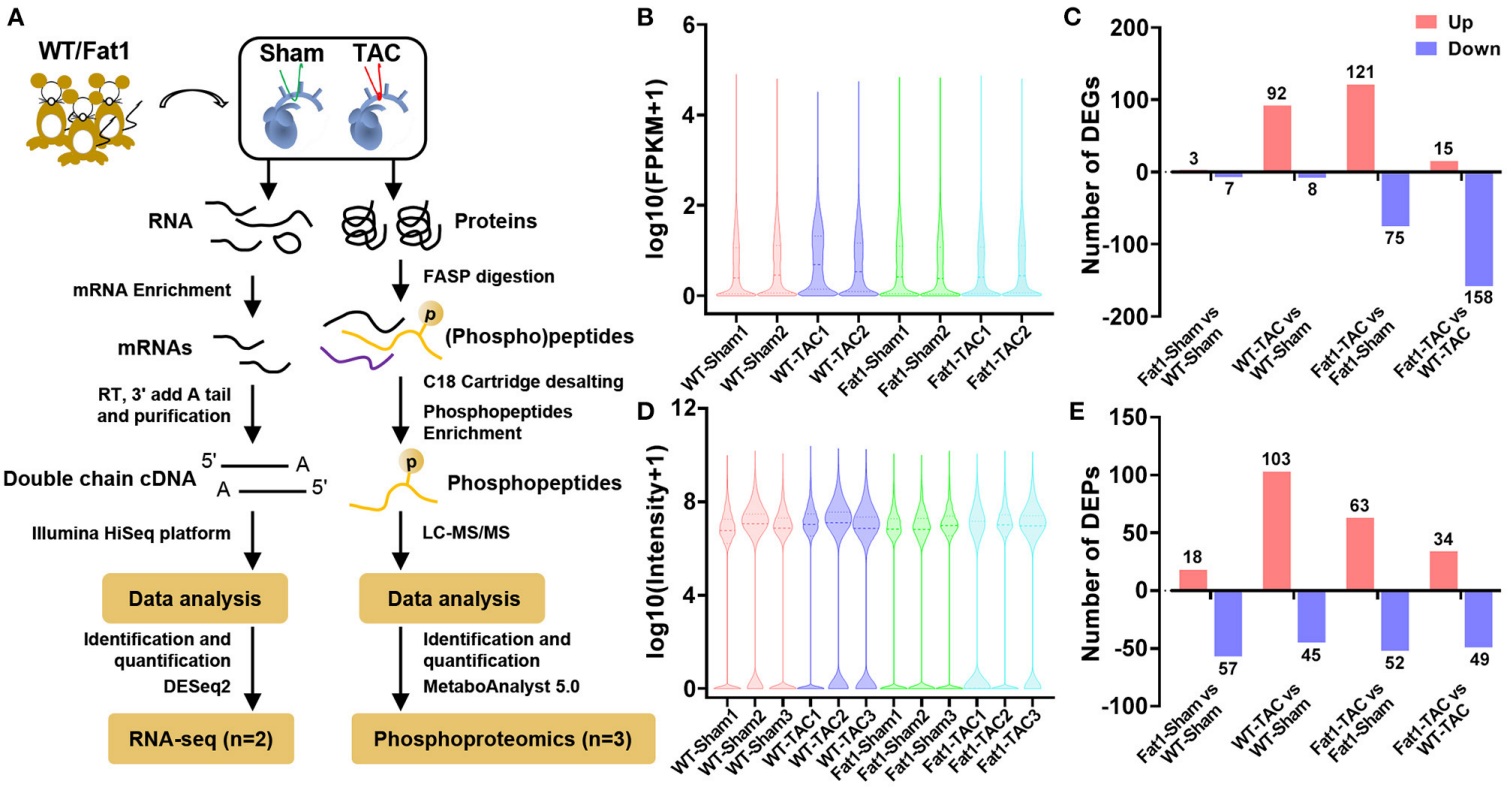
mRNA and phosphoprotein profiles. RNA-sequencing analysis of **(A)** overall mRNA expression profiles **(B)** and differentially expressed genes (DEGs) in pressure overload hearts **(C)**. Quantitative phosphoproteomic analysis **(A)** showed the overall expression profiles of phosphoproteins **(D)** and differentially expressed phosphoproteins (DEPs) in pressure overload hearts **(E)**. *N* = 2 and 3 for RNA-sequencing and phosphoproteomic analysis, respectively.

Transcriptomic and phosphoproteomic data analysis (Figure 2A) revealed the overall expression profiles of mRNAs and phosphoproteins, respectively (Figures 2B–E). Fat-1 transgene caused alterations (up- or downregulation) in the expression of mRNA under normal and pressure overload conditions (Figure 2C). Furthermore, analysis of the DEPs revealed that fat-1 transgene regulated expression of phosphoproteins during pressure overload. Specifically, compared with the WT hearts, the fat-1 hearts exhibited upregulation of 18 phosphoproteins and downregulation of 57 phosphoproteins. Meanwhile, under pressure overload conditions, fat-1 transgene up- and downregulated 34 and 49 phosphoproteins, respectively (Figure 2E).

**Figure 3:**
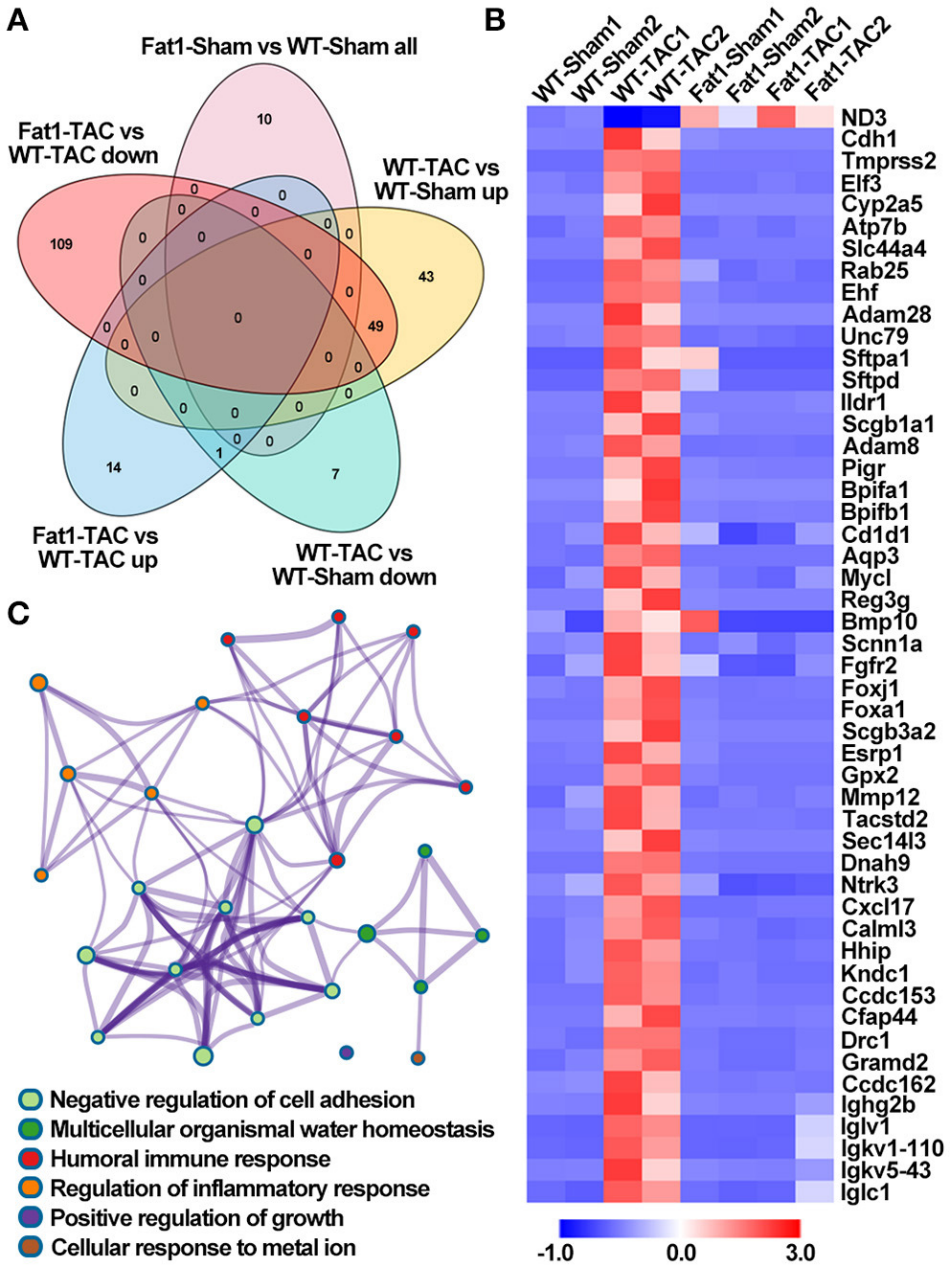
Effects of endogenous N-3 PUFAs on the expression of genes under pressure overload. Gene set enrichment analysis revealed that pressure overload significantly up-/down regulated the biological processes enriched in DEGs, which can be suppressed/restored by fat-1 transgene **(A,B)**. Under pressure overload conditions, elevated endogenous PUFAs downregulated these 49 genes enriched in six biological processes **(C)**. Fat1-Sham vs. WT-Sham all, genes upregulated and downregulated by fat-1 transgene; WT-TAC vs. WT-Sham up, genes upregulated by TAC-induced pressure overload; WT-TAC vs. WT-Sham down, genes downregulated by TAC-induced pressure overload; Fat1-TAC vs. WT-TAC up, genes downregulated by fat-1 transgene under pressure overload conditions; Fat1-TAC vs. WT-TAC down, genes downregulated by fat-1 transgene under pressure overload conditions. *N* = 2 for RNA-sequencing.

Gene set enrichment analysis of biological processes revealed that pressure overload significantly upregulated 49 genes (Figures 3A,B). Under pressure overload conditions, at the transcriptional level, elevated endogenous PUFAs downregulated these 49 genes enriched in cardiac remodeling (negative regulation of cell adhesion and positive regulation of growth), inflammation (humoral immune response and regulation of inflammatory response), and alterations in ion and cellular homeostasis (multicellular organismal water homeostasis and cellular response to metal ion) (Figures 3A,C).

**Figure 4:**
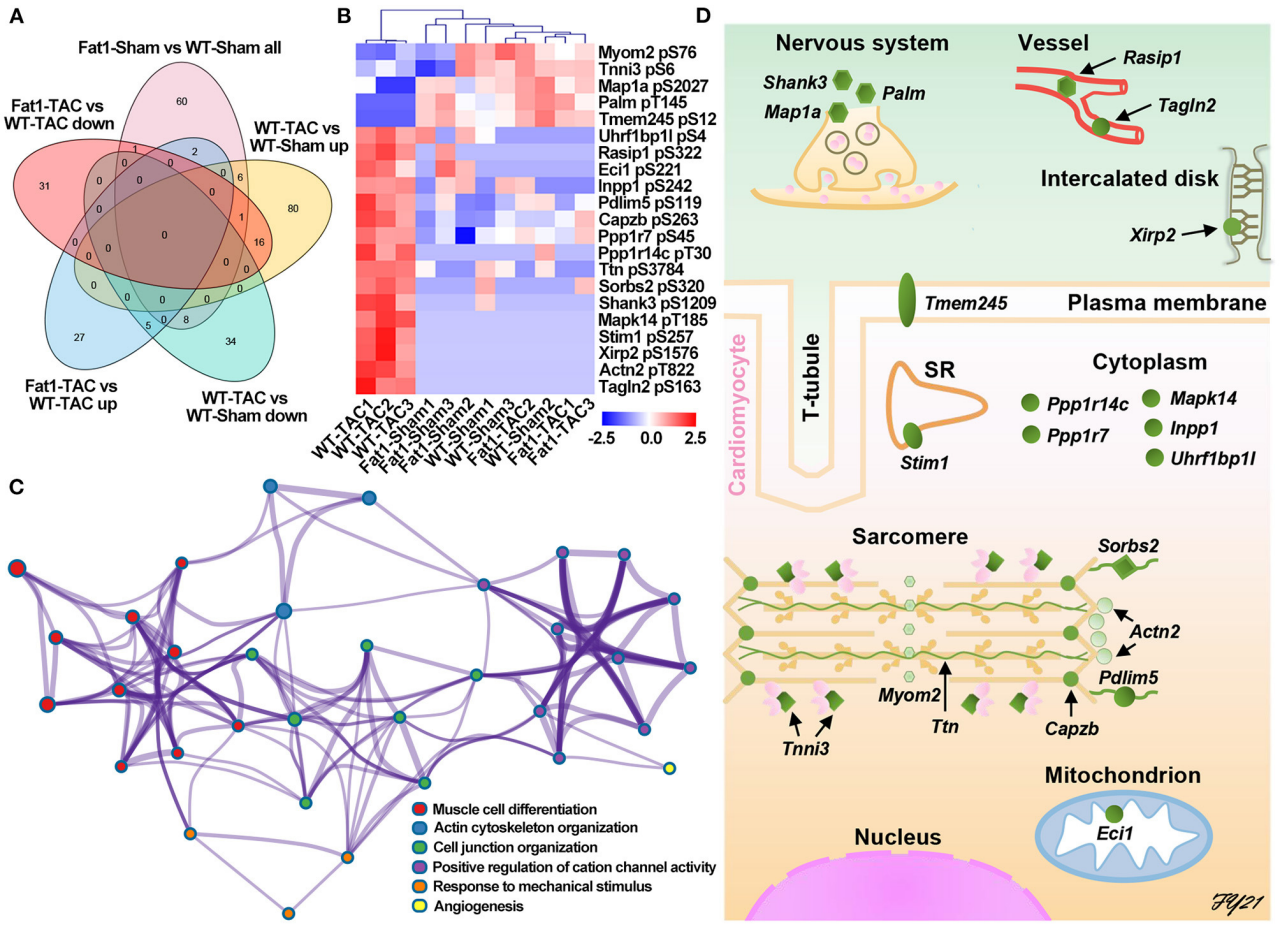
Protein phosphorylation affected by fat-1 transgene in pressure overload hearts. **(A)** Venn diagram analysis; **(B)** DEPs affected by fat-1 transgene under pressure overload conditions. **(C)** Elevated endogenous N-3 PUFAs suppressed phosphorylation signaling pathways that induce cardiac remodeling and alterations of ion hemostasis. **(D)** The location of each phosphoprotein is identified in the schematic illustration of the cardiomyocyte and its surrounding tissues, showing that endogenous n-3 PUFAs can function to plasma membrane, sarcomere reticulum (SR), cytoplasm, sarcomeres, and mitochondria of the cardiomyocytes, as well as the nerves and vessels. *N* = 3 per group.

We further analyzed protein post-translational modification using quantitative phosphoproteomics. Through Venn diagram analysis, we identified 21 phosphoproteins that were potentially affected by elevation of endogenous n-3 PUFAs (Figures 4A,B). Among them, five proteins were downregulated by pressure overload, but were upregulated by fat-1 transgene under pressure overload conditions, whereas another 16 proteins were upregulated by pressure overload, however, were downregulated by fat-1 transgene under pressure-overload (Table 2). Biological process enrichment for these 21 phosphoproteins revealed fat-1 transgene primarily suppressed expressions levels of phosphoproteins associated with cardiac remodeling (muscle cell differentiation, actin cytoskeleton organization, cell junction organization, angiogenesis) and maintained ion and cellular homeostasis (positive regulation of cation channel activity and response to mechanical stimulus) (Figure 4C). Moreover, these 21 phosphoproteins were localized within cardiomyocytes, nerves, and vessels in the myocardium, as well as in various subcellular compartments, including the plasma membrane, cytoplasm, sarcoplasmic reticulum, sarcomere, and mitochondria (Figure 4D; Table 2). Furthermore, phosphorylation of myofilaments was altered in dilated WT hearts (Figure 4D). Under pressure overload conditions, compared with WT mice, fat-1 mice showed higher phosphorylation levels of cTnI S6 and lower levels of titin S3784, β-actin capping protein S263, and α-actinin 2 T822 (Table 2; Supplementary Table S2). In addition, phosphorylation levels of two other Z-line proteins (PDZ and LIM domain protein 5, as well as sorbin and SH3 domain containing protein 2) were also restored by fat-1 transgene in pressure overload hearts. These results demonstrate that the contractile machinery represents one target of endogenous n-3 PUFAs in pressure overload hearts.

**Table 2:** Expression of phosphoproteins regulated by endogenous n-3 PUFAs.

**Location**	**Gene ID**	**Phosphoprotein**	**PO**	**Fat-1**
**Cardiomyocyte**				
Mitochondrion	Eci1	Enoyl-CoA delta isomerase 1, mitochondrial	**↑**	**↓**
Sarcomere	Tnni3	Cardiac troponin I	**↓**	**↑**
	Myom2	Myomesin 2	**↓**	**↑**
	Ttn	Titin	**↑**	**↓**
	Capzb	Actin capping protein, beta	**↑**	**↓**
	Actn2	Alpha actinin 2	**↑**	**↓**
	Pdlim5	PDZ and LIM domain protein 5	**↑**	**↓**
	Sorbs2	Sorbin and SH3 domain containing protein 2	**↑**	**↓**
Cytoplasm	Ppp1r14c	Protein phosphatase 1, regulatory inhibitor subunit 14C	**↑**	**↓**
	Ppp1r7	Protein phosphatase 1, regulatory subunit 7	**↑**	**↓**
	Mapk14	Mitogen-activated protein kinase, p38	**↑**	**↓**
	Inpp1	Inositol polyphosphate 1-phosphatase	**↑**	**↓**
	Uhrf1bp1l	UHRF1 binding protein 1-like	**↑**	**↓**
Intercalated disk	Xirp2	Xin actin-binding repeat containing protein 2	**↑**	**↓**
Sarcoplasmic reticulum	Stim1	Stromal interaction molecule 1	**↑**	**↓**
Plasma membrane	Tmem245	Transmembrane protein 245	**↓**	**↑**
**Nerve**				
	Palm	Paralemmin	**↓**	**↑**
	Map1a	Microtubule-associated protein 1A	**↓**	**↑**
	Shank3	SH3 and multiple ankyrin repeat domains 3	**↑**	**↓**
**Vessel**				
	Rasip1	Ras interacting protein 1	**↑**	**↓**
	Tagln2	Transgelin 2	**↑**	**↓**

*The phosphoproteins, which had been upregulated (downregulated) by pressure overload, were downregulated (upregulated) by endogenous n-3 PUFAs in pressure overload hearts. N = 3. ↑, upregulated; ↓, downregulated. PO, pressure overload, indicating protein expression affected by pressure overload; Fat-1, fat-1 transgene, indicating protein expression affected by fat-1 transgene under pressure overload condition*.

### Cardioprotection of Endogenous n-3 PUFAs Is Attributed to Suppression of Their Oxylipin Production in Pressure Overload Hearts

**Figure 5:**
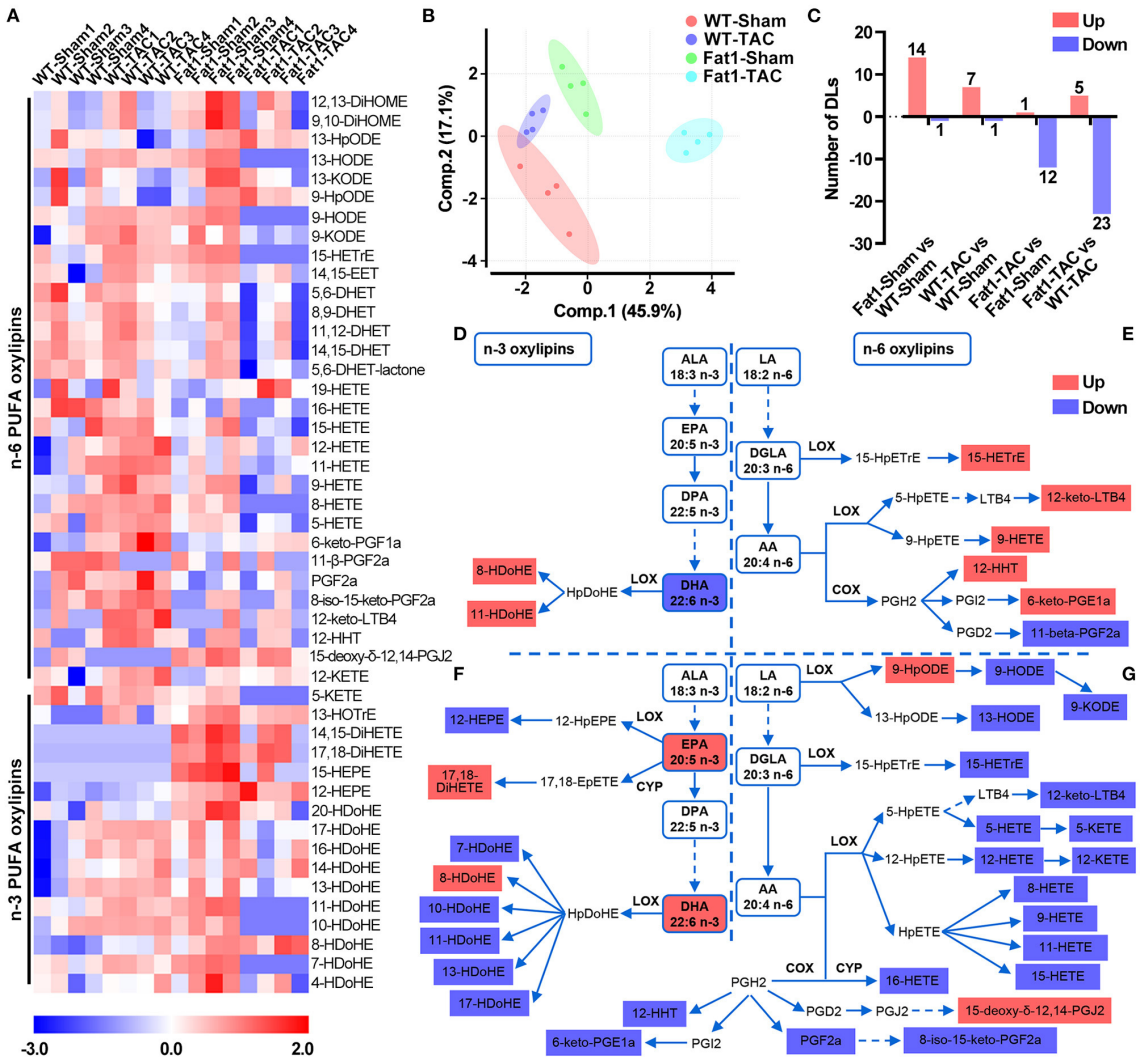
Signature of myocardial n-3/n-6 PUFA oxylipins in WT and fat-1 transgenic hearts under pressure overload conditions. Profile shifts of n-3 and n-6 oxylipins **(A)** were observed in the myocardium. The principal component analysis plot **(B)** showed that the altered profiles were distinct between groups, and the counts of altered oxylipins are shown in **(C)**. In the n-3 metabolic pathway, pressure overload reduced DHA levels but increased the levels of its oxylipins 8- and 11-HDoHE in the hearts of WT mice **(D)**. In the n-6 PUFA metabolic pathway, pressure overload increased the levels of 5 oxylipins, and decreased 1 oxylipin in the n-6 PUFA pathway **(E)**. Compared with WT hearts, the n-3 PUFA-protected hearts showed higher concentrations of EPA, DHA, and two oxylipins, whereas lower concentrations of 6 oxylipins were observed under pressure overload conditions **(F)**. Compared to WT hearts, fat-1 hearts increased the levels of 2 of oxylipins but decreased 18 oxylipins in the n-6 PUFA pathway **(G)**. *N* = 4 for each group.

We further explored the protective mechanisms of endogenous n-3 PUFAs based on lipidomic analysis. Shifts in the oxylipin profiles were observed in the myocardium with elevated n-3 PUFAs under pressure overload conditions (Figures 5A–C). Forty-seven oxylipins were detected, among which 16 belonged to n-3 and 31 belonged to n-6 metabolic pathways (Figure 5A). The number of oxylipins altered by pressure overload or fat-1 transgene is displayed in Figure 5C.

Analysis of the metabolic pathways (Figures 5D–G) showed that in the n-3 metabolic pathway, pressure overload reduced DHA levels but increased oxylipins 8- and 11-HDoHE levels in the hearts of WT mice (Figure 5D). Compared with WT hearts, the n-3 PUFA-protected hearts showed higher concentrations of EPA, DHA, and their oxylipins 17,18-DiHETE and 8-HDoHE, whereas lower concentrations of n-3 PUFA oxylipins (12-HEPE, and 7-, 10-, 11-, 13-, and 17-HDoHE) were observed under pressure overload conditions (Figure 5F). In the n-6 PUFA metabolic pathway, pressure overload increased the levels of n-6 PUFA oxylipins, including 15-HETrE, 12-keto-LTB4, 9-HETE, 12-HHT, and 6-keto-PGE1α and decreased the levels of the oxylipin 11-beta-PGF2α (Figure 5E). Compared to WT hearts, fat-1 hearts exhibited increased levels of n-6 PUFA oxylipins, including 9-HpODE and 15-deoxy-delta-12,14-PGJ2 and decreased levels of oxylipins in the LA-LOX pathway (9-HODE, 9-KODE, and 13-HODE), dihomo-γ-linolenic acid (DGLA)-LOX pathways (15-HETrE), AA-LOX pathways (12-keto-LTB4, 5-HETE, 5-KETE, 12-HETE, 12-KETE, 8-,9-,11-,15-HETE), AA-CYP pathway (16-HETE), and AA-COX pathway (12-HHT, 6-keto-PGE1α, PGF2α, and 8-iso-15-keto-PGF2α) (Figure 5G).

**Figure 6:**
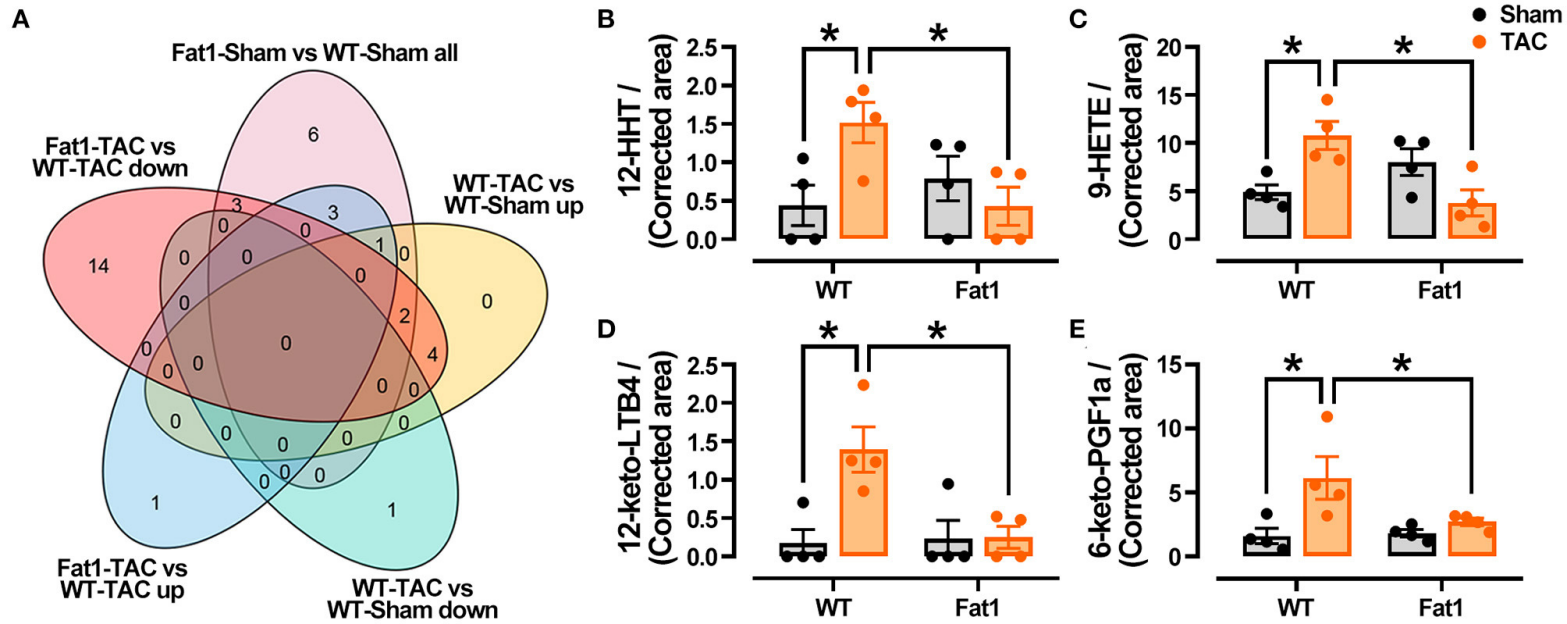
Fat-1 Oxylipin production in pressure overload hearts. **(A)** Venn diagram displaying the oxylipins that were decreased (increased) by pressure overload, were increased (decreased) by fat-1 transgene under pressure overload conditions. Four oxylipins including 12-HHT **(B)**, 9-HETE **(C)**, 12-keto-LTB4 **(D)**, and 6-keto-PGF1a **(E)** were identified via Venn diagram analysis. 12-HHT, 12(S)-hydroxyheptadeca-5Z,8E,10E-trienoic acid; 9-HETE, 9S-hydroxy-5Z,7E,11Z,14Z-eicosatetraenoic acid; 12-keto-LTB4, 12-keto-leukotriene B4; 6-keto-PGF1a, 6-keto-prostaglandin F1alpha. *N* = 4 for each group. ^*^ *P* < 0.05 between groups.

Furthermore, after analyzing differences in oxylipin production within the myocardia (Figure 6A), we found that four oxylipins, namely, 12-HHT, 9-HETE, 12-keto-LTB4, and 6-keto-PGF1α were significantly reduced in the pressure overload myocardium protected by n-3 PUFAs (Figures 6B–E). The oxylipin profiles in the Fat1-TAC hearts revealed that protective effects of endogenous n-3 PUFAs in pressure overload hearts were attributed to their oxylipins.

### Endogenous n-3 PUFAs Protect Mitochondrial Structure and Function Under Pressure Overload Conditions

**Figure 7:**
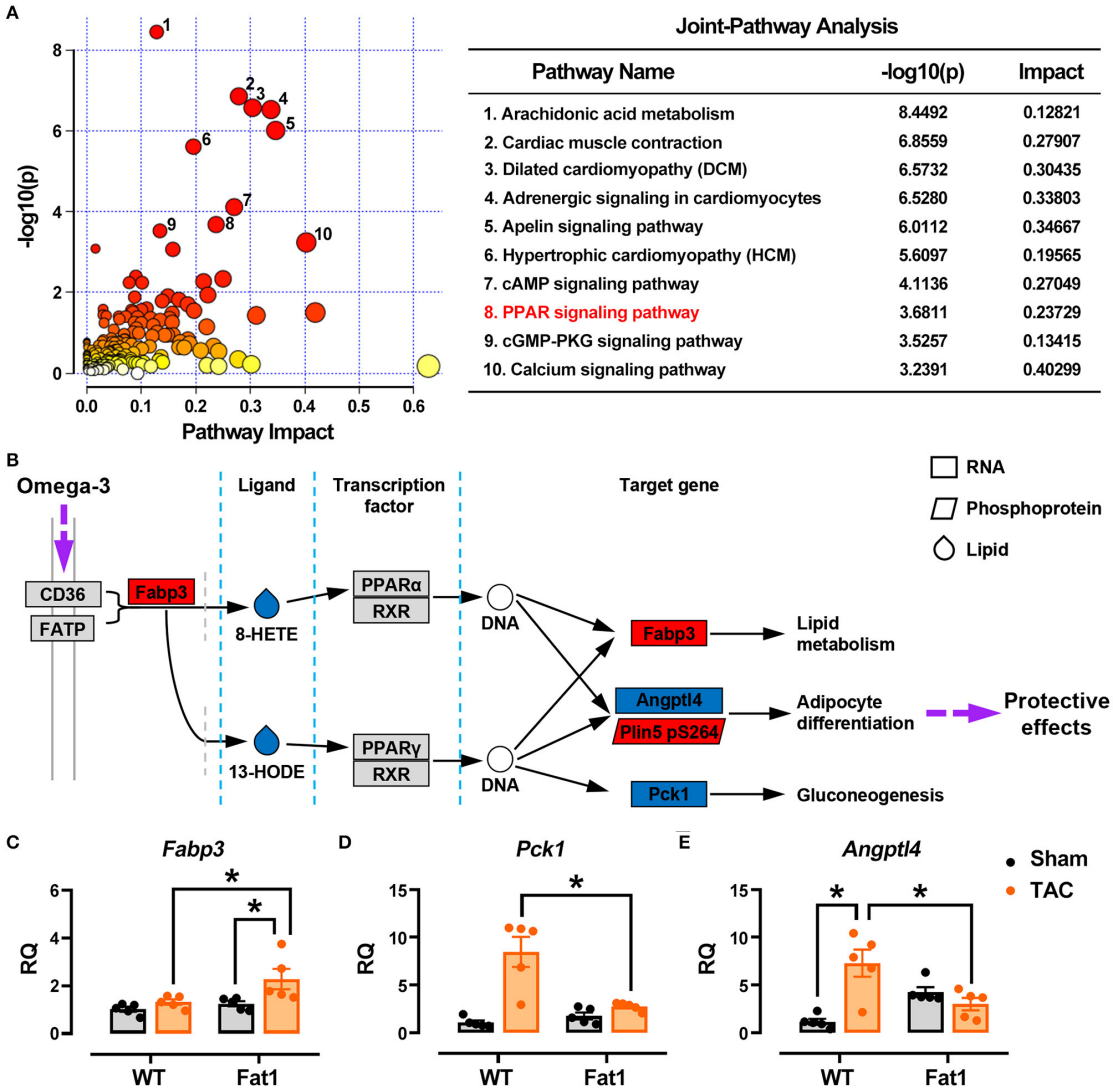
Multi-omics data analysis. The results of RNA-sequencing, phosphoprotein quantification, and oxylipin profiling were integrated and analyzed **(A)**. Top 10 pathways affected by endogenous n-3 PUFAs were selected for analysis, and KEGG pathway analysis was applied to illustrate protective mechanism of n-3 PUFAs in pressure overload hearts **(B)**. Expression of genes identified from multi-omics data analysis has been determined by RT-qPCR **(C–E)**. ^*^ *P* < 0.05 between groups.

The results of RNA-sequencing, phosphoprotein quantification, and oxylipin profiling were integrated and analyzed (Figure 7A). The top 10 enriched pathways were involved in metabolism (arachidonic acid metabolism, apelin signaling pathway, peroxisome proliferator-activated receptors (PPAR) signaling pathway, and cGMP-PKG signaling pathway), contractile function (cardiac muscle contraction, adrenergic signaling in cardiomyocytes, cAMP signaling pathway, and calcium signaling pathway), cardiac remodeling (dilated cardiomyopathy and hypertrophic cardiomyopathy). Furthermore, we found that fat-1 transgene altered PPAR signaling pathway, a metabolic pathway links the genes-phosphoproteins-metabolite regulatory network. We compared the profiles of genes, proteins and metalates with PPAR signaling pathway–Mus musculus (mmu03320) of the KEGG database (https://www.genome.jp/kegg-bin), and found that three genes (*Fabp3, Pck1*, and *Angptl4*), one phosphoprotein (*Plin5* encoding PDZ and LIM domain protein 5), and two oxylipins (8-HETE, and 13-HODE) matched the pathway (Figure 7B). Gene expression verification showed that *Fabp3* was upregulated, but *Pck1* and *Angptl4* were downregulated by fat-1 transgene under pressure overload conditions (Figures 7C–E). The KEGG pathway analysis further supported that endogenous n-3 PUFAs protect hearts through regulating gene expression, protein phosphorylation and lipid metabolism.

**Figure 8:**
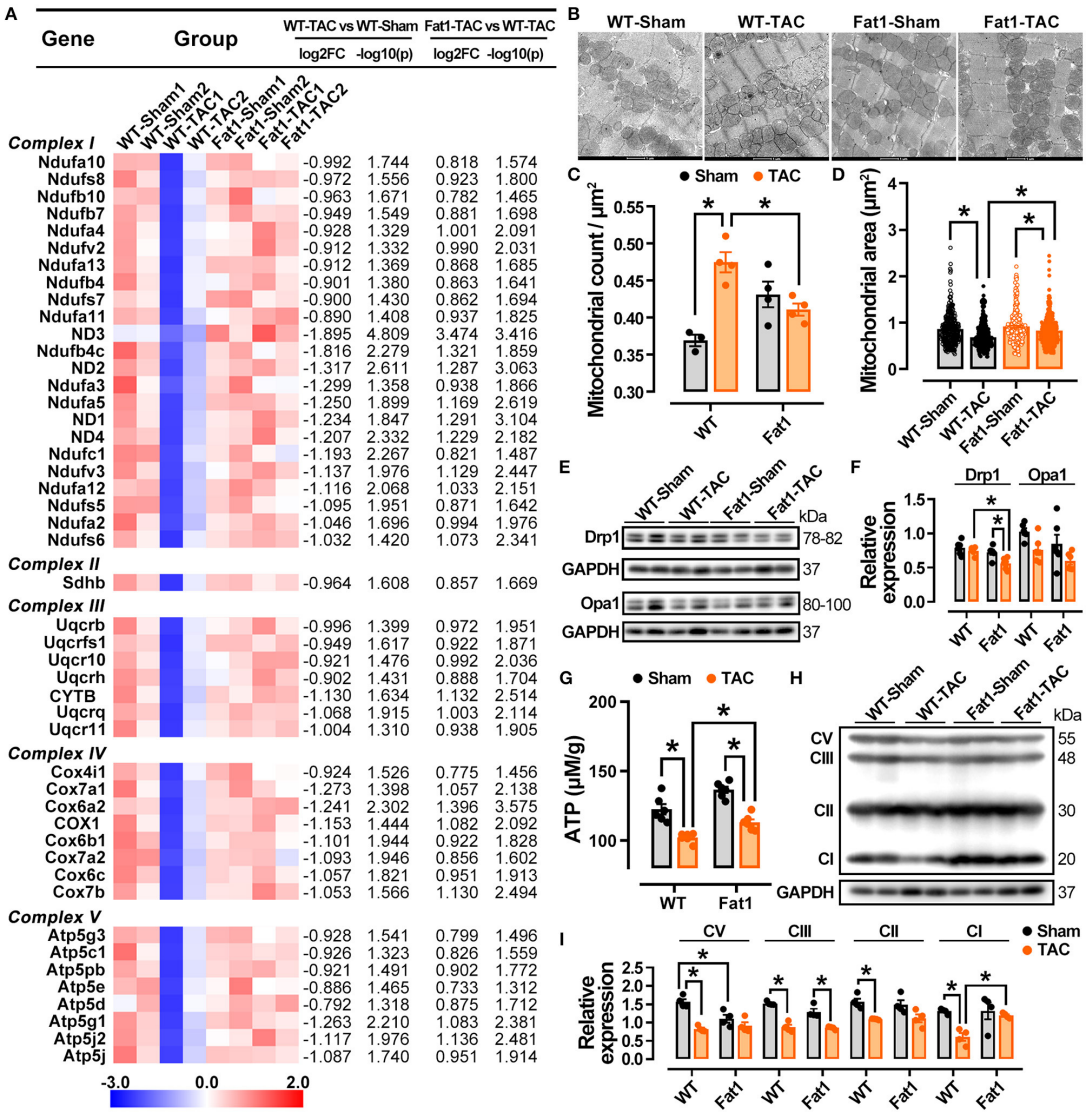
Preservation of mitochondria function by fat-1 transgene in pressure overload hearts. In the RNA-sequencing datasets of experimental groups, 47 genes encoding oxidative phosphorylation markers, mitochondrial respiratory chain complexes were showed **(A)**. Under transmission electron microscopy, the mitochondria display a reduction in size and increase in counts per square μm in WT-TAC hearts, whereas those of Fat1-TAC hearts were not altered by pressure overload **(B–D)**, *N* = 300 mitochondria in the measurement area. The fission marker Drp1 was significantly reduced in Fat-1 TAC hearts but not in the dilated WT-TAC heart **(E,F)**. ATP generation was restored by endogenous n-3 PUFAs under pressure overload conditions **(G)**. The protein expression of respiratory chain complexes was downregulated in the dilated WT-TAC hearts. Compared to the sham hearts, pressure overload did not suppress the expression levels of mitochondrial complexes I, II, V in fat-1 transgenic hearts **(H,I)**. *N* = 4 for each group. ^*^P < 0.05.

Mitochondrial function associated RNA-sequencing datasets was examined. Expression of 47 genes encoding oxidative phosphorylation markers, specifically mitochondrial respiratory chain complexes, were significantly downregulated by pressure overload (fold-change > 1.5 and *P* < 0.05). Among them, 23, 1, 7, 8, and 8 genes belonged to complexes I, -II, -III, -IV, and -V, respectively. However, elevation of endogenous n-3 PUFAs restored their expression (Figure 8A). Under TEM, the mitochondria were reduced in size and more abundant per square μm in WT-TAC hearts, whereas the mitochondria of Fat1-TAC hearts were normal in terms of shape and size (Figures 8B–D). Western blotting analysis further revealed that expression of the fission marker Drp1 was significantly reduced in fat-1 TAC hearts but not in the dilated WT-TAC heart (Figures 8E,F). These results confirm that endogenous n-3 PUFAs protected hearts from mitochondrial injury under the pressure overload condition.

Consistent with the protection of mitochondrial structure in fat-1 TAC hearts, the ATP generation was also restored by endogenous n-3 PUFAs under pressure overload conditions (Figure 8G). Furthermore, by examining the protein expression of complexes I, -II, -III, and -V, we found that all complexes were downregulated in the dilated WT-TAC hearts. Compared to in the sham hearts, TAC did not suppress the expression of mitochondrial complexes. In contrast, the expression of complex V was significantly upregulated in fat-1 TAC hearts (Figures 8H,I). These results demonstrate that endogenous n-3 PUFAs regulate mitochondrial oxidative phosphorylation and ameliorate mitochondrial dysfunction under pressure overload conditions.

## Discussion

In the present study, we explored the protective mechanisms of n-3 PUFAs in DCM. This is the first study to employ quantitative phosphoproteomics and multi-omics data analysis to reveal the protective mechanisms provided by endogenous n-3 PUFAs in DCM. Our results reveal that elevated endogenous n-3 PUFAs reduce cardiac remodeling and improve cardiac function. Under pressure overload conditions, n-3 PUFAs target phosphorylation signaling and restore the phosphorylation states of intracellular proteins in the subcellular compartments of sarcomeres, cytoplasm, membranes, sarcoplasmic reticulum, and mitochondria. Further mechanistic studies revealed that endogenous n-3 PUFAs suppress the production of detrimental n-3/n-6 oxylipins and regulate a metabolic associated gene-protein-metabolite pathway. Specifically, the mitochondrial structure, dynamics, oxidative phosphorylation, and function were protected by endogenous n-3 PUFAs in pressure overload hearts. Collectively the results provide insights into the protective mechanisms of n-3 PUFAs in DCM, which involve multiple cellular processes (transcription, post-translational modification, cellular metabolism, mitochondrial oxidative phosphorylation, and mitochondrial dynamics and function) in various cellular compartments. Specifically, our findings demonstrate that mitochondria play a critical role in n-3 PUFA-dependent cardioprotection in DCM.

### N-3 PUFAs Maintain Phosphorylation States of Proteins and Prevent Cardiac Remodeling

We employed a transgenic mouse model expressing the *C. elegans* gene fat-1 to explore the protective mechanisms of n-3 PUFAs. Previous studies have employed this transgenic model to reveal that n-3 PUFAs exert anti-inflammatory and anti-apoptotic roles under various conditions (28–30). In addition, n-3 PUFAs reportedly increase phospholipids under hypertensive conditions in postnatal rats (31). Although supplementation with n-3 PUFAs is widely performed by individuals, evidence of the effects of n-3 PUFA treatment from clinical trials is inconsistent, and the molecular mechanisms underlying their effects remain unclear (32). Consistent with the evidence reported by Endo et al. (28), we found that elevated endogenous n-3 PUFAs prevents cardiac remodeling induced by pressure overload. In addition, the structural protection, genes and phosphoproteins enriched in cardiac remodeling were reduced by elevation of endogenous n-3 PUFAs. Activation of multiple phosphorylation signaling pathway is associated with cardiac hypertrophy or dilation. Several kinases such as AMP-activated protein kinase and protein kinase C have been recognized as valuable targets for drug discovery (33, 34). Here, we observed that endogenous n-3 PUFAs induced phosphorylation of proteins in the cardiomyocytes, nerves, and vessels of the heart. Modification of these proteins was centered in the regulation of contractile function. Among the 16 differentially abundant phosphoproteins in the cardiomyocytes, nine interacts with the myofilaments, phospholamban complex, and sarcomeric proteins at the intercalated disks (22, 35, 36), while four cytosolic proteins target either sarcomere or excitation-contraction coupling (37). Previous phosphoproteomic studies have provided insights regarding the roles of myofilament phosphorylation in cardiac contractile function. For instance, cTnI phosphorylated at Ser23/Ser24 regulates the myofilament response to Ca^2+^ (38) and is significantly increased in heart failure. Herein, we found that fat-1 transgene suppresses cTnI S23 phosphorylation in pressure overload hearts (Supplementary Table S1). Another phosphorylation site of cTnI, involving the serine 6 (S6) residue may also play a key role in mediating the beneficial effects of n-3 PUFA in fat-1 hearts during DCM. The phosphorylation level of cTnI S6 was downregulated in dilated hearts, and upregulated in fat-1 hearts under pressure overload conditions. Zhang et al. (39) further demonstrated that pseudo-phosphorylation (S6D) in myofilaments depresses the maximum tension of skinned muscle fibers (39). Taken together, elevation of myocardial n-3 PUFA can be a valuable strategy to maintain phosphorylation-dependent regulation of contractile function under pressure overload conditions. However, further studies are needed to clarify the roles of phosphorylation of sarcomeric or cytosolic proteins that are regulated by n-3 PUFAs in DCM.

Post-translational modification of cytosolic proteins involving p38, protein phosphatase type 1, and phosphatidylinositol signaling pathways, contribute to the protective myocardial effect provided by endogenous n-3 PUFAs. These protein kinases and phosphatases influence multiple cardiac activities and are essential for maintaining the hemostasis of cardiomyocytes. For instance, phosphorylation of p38 a key regulator in cardiac development and regeneration (40, 41), while its phosphorylation is decreased in failing human cardiomyocytes (42). Here, we observed an increase in the phosphorylation p38 T185 by pressure overload in WT hearts, and upregulated phosphorylation of p38 T185 in the dilated hearts was restored in fat-1 hearts, suggesting that this residue of p38 can be crucial to functional adaptation for the stressed hearts. In summary, alterations in post-translational modifications of the signaling or structural proteins in the myocardium contribute to the cardioprotective effect of n-3 PUFAs in DCM.

### N-3 PUFAs Protect Mitochondrial Structure, Dynamics, and Function

The protection of mitochondria in pressure overload-induced DCM has been observed to involve several processes, including restoration of the expression of mRNAs encoding mitochondrial proteins, preservation of mitochondrial oxidative phosphorylation, maintenance of the mitochondrial structure, control of the phosphorylation levels of mitochondrial proteins, and regulation of mitochondrial dynamics and function. N-3 PUFAs are involved in both enzymatic and non-enzymatic n-3 oxidation pathways (32). In the enzymatic oxidation pathway, phospholipase A2 activates PLA2 and releases EPA or DHA. Next, they are catalyzed by COX, LOX, and CYP enzymes to form a series of oxylipins, including PGs and TXAs. In the non-enzymatic oxidation pathway, reactive oxygen species oxidize EPA and DHA and form lipid peroxides of EPA and DHA, which further metabolize into isoprostanes, isoflurane, alkenes, etc. In this study, we identified several n-3 and n-6 oxylipins that were upregulated in dilated WT hearts. Some of these metabolites are associated with cardiovascular dysfunction. For example, plasma HDoHEs, which are oxidative stress indicators, are elevated in hypertensive patients (43). Moreover, plasma HETEs, including 5-, 8-,9-, 11-, 12-, 15-, are upregulated in patients with acute coronary syndrome (44). Meanwhile, mechanistic studies have revealed that 8-HETE and 15-HETE induce hypertrophy in a cardiomyocyte cell line and pulmonary hypertension in rats, respectively (45, 46). Consistent with these findings, we found that the myocardia protected by endogenous n-3 PUFAs exhibited reduced levels of most n-3 (6 in 8 oxylipins) and n-6 oxylipins (18 in 20 oxylipins). Thus, shifts in the metabolic profiles of oxylipins may protect cardiac function by assisting the fat-1 myocardia to resist pressure overload stress.

Furthermore, our integrated multi-omics data revealed that endogenous n-3 PUFAs regulated the expression of various genes and phosphoproteins, as well as the metabolism of oxylipins through the PPAR pathway in pressure overload myocardia. The effectors of oxylipins included PPAR-regulated genes *Fabp3* (upregulated), *Angptl4* (downregulated*)*, and *Pck1* (downregulated) and phosphoprotein perilipin 5. In healthy hearts, PPAR isoforms are activated by fatty acids and act as a master switch in controlling cardiomyocyte metabolism (47). Meanwhile, PPAR agonists are used for treating diabetes and hyperlipidemia, which are risk factors for incident heart failure (48). In this study, we found that fat-1 transgene targeted 8-HETE and 13-HODE and in turn suppressed *Angptl4* and *Pck1* to fine tune lipid and glucose metabolism in the pressure overload hearts. *Angptl4* encodes angiopoietin-like 4 protein, which is a key regulator of lipoprotein lipase, and *Pck1* encodes phosphoenolpyruvate carboxykinase 1, which catalyzes gluconeogenesis. However, the roles of these molecular signals in the stressed heart remain unclear. For example, upregulation of *Angptl4* has been reported in patients with, and an experimental model of, metabolic syndrome (49, 50), whereas activation of cardiac Angptl4 via PPARβ/δ is associated with cardioprotection under lipid overload conditions (51). In addition, We found that phosphorylation of perilipin 5 is associated with the cardiac protective effect in pressure overload fat-1 hearts. Super-resolution microscopy has shown that perilipin 5 is localized at lipid droplet-mitochondria tethering sites (52), suggesting that perilipin 5 is crucial to mitochondrial function. Moreover, activation of perilipin 5 has been shown to protect hearts against oxidative stress and ischemia (53, 54). However, the regulation and function of this gene-metabolite-phosphoprotein signaling network requires further investigation.

Myocardium ATP contents were restored by fat-1 transgene in pressure overload hearts, confirming that n-3 PUFAs protect cardiac metabolic and contractile machinery. Furthermore, we found that mitochondrial oxidative phosphorylation was maintained by fat-1 transgene in pressure overload myocardia. More than 95% of the energy used by cardiomyocytes is produced by oxidative phosphorylation (55) with ~60–70% of the total ATP produced used to fuel cardiomyocyte contraction, while the remainder is used for various ion pumps including CaATPase in the sarcoplasmic reticulum (56). Reportedly, in heart failure, oxidative phosphorylation levels are reduced (57). Consistent with this finding, we found that pressure overload-induced dilation significantly reduced expression of respiratory chain complexes I–V, whereas myocardia with elevated endogenous n-3 PUFAs resisted the pressure overload and retained the expression of respiratory chain complex I, -II, and -IV. However, in contrast to the above findings, the mRNA profiling of the respiratory chain complex was preserved in the pressure overload fat-1 hearts.

In conclusion, endogenous n-3 PUFAs prevent dilated cardiomyopathy via orchestrating gene expression, protein phosphorylation, and metabolism. The functional protection of n-3 PUFAs in pressure overload hearts was attributed to preservation of mitochondrial structure, dynamics, and function.

### Limitations of the Study

Illustration of the molecular signatures allows us to explore the mechanisms and evaluate therapeutic effects in an unbiased way. However, due to the limitations of research tools, molecular changes detected in unbiased screening of small populations may be difficult to verify in large-scale populations. For example, no commercially available antibodies exist for cTnI T6. In addition, quantitative proteomics indicated that 21 phosphoproteins were mediated by n-3 PUFAs in pressure overload hearts; However, whether these molecules represent specific determinants has not yet been verified, and warrants further investigation to fully elucidate the associated molecular mechanisms.

## Data Availability Statement

The datasets presented in this study can be found in online repositories. The names of the repository/repositories and accession number(s) can be found at: GEO, GSE185686.

## Ethics Statement

The animal study was reviewed and approved by Institutional Animal Care and Use Committee (IACUC) of the Guangdong Laboratory Animals Monitoring Institute (No. IACUC2017015).

## Author Contributions

FY, XL, and WT designed and initiated the project. WT performed the animal surgery and echocardiographic recording. CC and JZ performed the lipidomic analysis. XL, SZ, HC, CY, LK, and ZP were responsible for western blotting, imaging measurement, pathological examination, or animal care. XL and FY performed the bioinformatics analysis, interpreted the results, and prepared the manuscript. YZ, CZ, WP, and PB provided critical comments during experiment design and manuscript preparation. All authors read and approved the final manuscript.

## Funding

This work was supported by the Guangzhou Science and Technology Program (201804010206), the Guangdong Science and Technology Program (2018A0303130208, 2019A030317014, and 2008A08003), National Natural Science Foundation of China (31672376, 81941002), and the Guangdong Provincial Key Laboratory of Laboratory Animals (2017B030314171). WP is supported with a Senior Investigator Award for Women's Heart Health from the Heart and Stroke Foundation of Canada.

## Conflict of Interest

The authors declare that the research was conducted in the absence of any commercial or financial relationships that could be construed as a potential conflict of interest.

## Publisher's Note

All claims expressed in this article are solely those of the authors and do not necessarily represent those of their affiliated organizations, or those of the publisher, the editors and the reviewers. Any product that may be evaluated in this article, or claim that may be made by its manufacturer, is not guaranteed or endorsed by the publisher.

## Abbreviations

ALA: alpha lipoic acid
DHET: dihydroxyeicosatrienoic
DiHETE: dihydroxyeicosatetraenoic acid
DiHOME: Dihydroxy octadecenoic acid
DPA: docosapentaenoic acid
EET: epoxyeicosatrienoic acid
GLA: gamma linolenic acid
HDoHE: hydroxydocosahexaenoic acid
HETE: hydroxyeicosatetraenoic acid
HETrE: hydroxy eicosatrienoic acid
HEPE: hydroxyeicosapentaenoic acid
HHT: hydroxyheptadecatrenoic acid
HODE: hydroxy octadecadienoic acid
HOTrE: hydroxyoctadecatrienoic acid
HpODE: hydroperoxylinoleic acids
KETE: ketoeicosatetraenoic acid
KODE: ketooctadecadienoic acid
LA: linoleic acid
LTX: leukotriene X
PGX: prostaglandin X.
