# Supplementary material for: Cardioprotective Effects of n-3 Polyunsaturated Fatty Acids: Orchestration of mRNA Expression, Protein Phosphorylation, and Lipid Metabolism in Pressure Overload Hearts
Source: Front Cardiovasc Med. 2022 Jan 3;8:788270. doi: 10.3389/fcvm.2021.788270 (PMC8761763; doi:10.3389/fcvm.2021.788270)
Supplement: Supplementary file 1 [file Data_Sheet_1.pdf]

Supplementary Material

Cardioprotective effects of n-3 polyunsaturated fatty acids: Orchestration of mRNA expression, protein phosphorylation, and lipid metabolism in pressure overload hearts

Xiang Li[1#], Weijiang Tan[1,2#], Shuang Zheng[1#], Junjie Zhang[3#], Caiyi Zhu[1], Chun Cai[3], Honghua Chen[1], Chenqi Yang[4], Le Kang[5], Zhanhong Pan[1], W. Glen Pyle[6], Peter H. Backx[7,8], Yunzeng Zou[5], Feng Hua Yang[1*]

Supplemental Table S1. RT-qPCR Primers used in this study.

Gene	Forward	Reverse
**Pck1**	ATTCAACGCCAGGTTCCCAG	GCCTCCAGCACAGATATGCC
**Fabp3**	TGGAAGCTAGTGGACAGCAAG	CCCCGTTCTTCTCGATGATGG
**Angpt14**	AACGCCACCCACTTACACAG	TGAAGTCCACAGAGCCGTTC
**β-actin**	GATATCGCTGCGCTGGTCG	CATTCCCACCATCACACCCT

Supplemental table S2. Phosphoproteins were significantly up-/downregulated by fat-1 expression under pressure overload conditions. N=3 for each group.

**Upregulated**					
Protein (Gene ID)	log2(FC)	P-Value	Protein (Gene ID)	log2(FC)	P-Value
Mecp2 pS80	4.9589	0.016627	Plin5 pS264	2.517	0.000038703
Slc2a4 pT486	4.1358	0.010688	Hnrnpu pS4	2.5148	0.045941
Tns1 pS1182	3.7514	0.0043428	Tmem245 pS12	2.4712	0.000013447
Ppp1r14c pS33	3.5537	0.0031806	Sfr1 pT54	2.4536	7.1604E-06
Palm pT145	3.4509	0.0025533	Rcan3 pS105	2.4445	6.6457E-06
Scn7a pT57	3.2435	0.0017683	Faf1 pT268	2.3824	0.044442
Atp2a2 pT441	3.1914	0.0013348	Sptb pS36	2.3814	2.0127E-07
Hrc pS151	3.1335	0.0020992	Kank3 pS113/S144	2.2879	0.024871
Ttn pT24135	3.053	0.00087779	Plec pS1572	2.2106	0.042069
Rere pS332	3.0405	0.01672	Map1a pS2027	2.0363	0.047186
Trim28 pS23	3.0329	0.011847	Carhsp1 pS53	1.8055	0.034071
Srrm2 pS1278	3.0063	0.00070822	Tnni3 pS6	1.6642	0.0082615
Itpr1 pS1588	2.8744	0.00058418	Myom2 pS76	1.5444	0.020717
Sync pT315	2.7298	0.00054887	Hacd3 pS114	0.97051	0.0055777
Hbbt1 pS53	2.6857	0.017145	Mtfr1l pS100	0.65687	0.043216
Actn2 pT825	2.6825	0.00034163	Stom pS161	0.61439	0.018526
Ckm pS372	2.6608	0.029722			
**Downregulated**					
Protein (Gene ID)	log2(FC)	P-Value	Protein (Gene ID)	log2(FC)	P-Value
Pdlim5 pS119	-0.62692	0.030605	Hrc pS421	-2.6981	0.040257
Slc2a4 pS10	-0.75368	0.038092	Mapk14 pT185	-2.8624	0.00046692
Ptpn12 pS673	-0.77691	0.041639	Rasip1 pS322	-2.905	0.00081357
Bckdha pS334	-0.86336	0.035769	Tagln2 pS163	-2.9695	0.0031424
Ablim1 pT454	-1.1323	0.0041777	Mical3 pS685	-3.0867	0.003061
Ryr2 pT2809	-1.199	0.0068779	Ndufb6 pY4	-3.1045	0.0014401
Jph1 pS448	-1.2534	0.0029331	Pacsin3 pS354	-3.1188	0.035366
Mindy1 pS27	-1.3054	0.031489	Tns2 pS830	-3.2075	0.0068715
Ppp1r7 pS45	-1.485	0.036798	Tnni3 pS23	-3.2154	0.030294
Capzb pS263	-1.7215	0.034041	Svil pS510	-3.2321	0.0040967
Cttn pT364	-1.7956	0.046207	Fundc2 pS5	-3.2489	0.0016369
Inpp1 pS242	-1.9065	0.038618	Actn2 pT822	-3.2762	0.0017554
Larp1 pS498	-1.9846	0.049627	Exoc1 pS458	-3.297	0.0018487
Ttn pS3870	-2.0341	0.048235	Pdcd5 pS119	-3.3098	0.029436
Serbp1 pS284	-2.2347	0.042834	Kank3 pT239	-3.3606	0.0076365
Pea15 pS116	-2.3638	0.031149	Rev3l pS758/S761/S763/S766	-3.3633	0.0043089
Sorbs2 pS320	-2.3759	0.025361	Ppp1r14c pT30	-3.3666	0.0026704
Palmd pS372	-2.4085	6.8373E-07	Stim1 pS257	-3.3882	0.0037663
Myh6 pS1896	-2.4199	0.029579	Uhrf1bp1l pS4	-3.4458	0.0040488
Ttn pS3784	-2.4471	2.4336E-06	Xirp2 pS1576	-3.7469	0.0061821
Chd4 pS1515	-2.6169	0.000076826	Tfip11 pS60	-3.8713	0.0066954
Tns1 pS1578	-2.6634	0.000082438	Shank3 pS1209	-4.3381	0.0086362
Rer1 pS95	-2.6635	0.00050472	Eci1 pS221	-4.346	0.0088948
